# Antimicrobial and Antioxidant Effects of Aqueous, Methanolic, and a Deep Eutectic Solvent–Based Extracts of *Althaea officinalis*

**DOI:** 10.1155/sci5/9161837

**Published:** 2026-02-25

**Authors:** Hossein Khodadadi, Reza Ghasemi, Forough Karami, Ahmad Vaez, Hasti Nouraei, Kimia Sahraeian, Zahra Zareshahrabadi

**Affiliations:** ^1^ Department of Medical Parasitology and Mycology, Shiraz University of Medical Sciences, Shiraz, Iran, sums.ac.ir; ^2^ School of Medicine, Student Research Committee, Shiraz University of Medical Sciences, Shiraz, Iran, sums.ac.ir; ^3^ Central Research Laboratory, School of Medicine, Shiraz University of Medical Sciences, Shiraz, Iran, sums.ac.ir; ^4^ Department of Tissue Engineering and Applied Cell Sciences, Shiraz University of Medical Sciences, Shiraz, Iran, sums.ac.ir; ^5^ Basic Sciences in Infectious Diseases Research Center, Shiraz University of Medical Sciences, Shiraz, Iran, sums.ac.ir

**Keywords:** *Althaea officinalis*, antibacterial, antifungal, antioxidant, deep eutectic solvent

## Abstract

**Background:**

The increase in antimicrobial resistance has become a worldwide health emergency, rendering most conventional antibiotics ineffective and encouraging the research into alternative therapeutic methods.

**Methods:**

The antioxidant and antimicrobial activity of *Althaea officinalis* flower extracts was investigated in this research with specific focus on the deep eutectic solvent–mediated extraction method. Deep eutectic solvent was synthesized using ammonium acetate and lactic acid in different molar ratios and utilized as a sustainable extraction solvent under ultrasound‐assisted extraction conditions. Deep eutectic solvent–based extraction was optimized to produce high amounts of bioactive compounds, and extracts obtained were compared with aqueous and methanolic solvents. Total phenolic content, antimicrobial activity against standard bacterial and fungal strains, as well as azole‐resistant and azole‐sensitive clinical isolates of *Candida albicans*, were measured. Antioxidant capacity was calculated by 2,2‐diphenyl‐1‐picrylhydrazyl radical scavenging assay.

**Results:**

Deep eutectic solvent–based extract was superior in extraction with greater total phenolic content (8.9 ± 0.4) and improved antimicrobial and antioxidant activity compared to the conventional extracts. Particularly, the deep eutectic solvent extract exhibited noteworthy inhibitory activity against Gram‐positive and Gram‐negative standard strains of bacteria, as well as standard and clinical yeast fungi. The biocompatibility of the deep eutectic solvent extract was assured by cytotoxicity analyses against 3T3 fibroblast cells.

**Conclusions:**

These results demonstrate that DES‐based extraction serves as a green and effective method for isolating bioactive compounds from *Althaea officinalis*. This procedure deserves its potential use in the synthesis of novel antimicrobial agents.

## 1. Introduction

**Figure 1:**
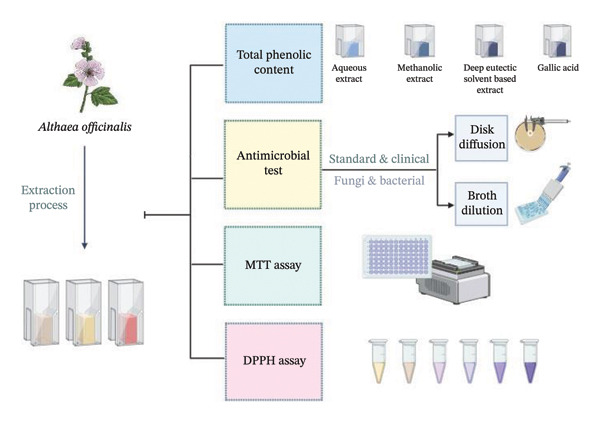
Schematic overview of the research workflow. The figure provides a graphical summary of the study’s conceptual outline and experimental approach. The diagram summarizes the main hypothesis, key methodological steps, and anticipated outcomes.

The rise of antimicrobial resistance has become a worldwide health emergency, rendering most conventional antibiotics ineffective and motivating the research for alternative therapeutic methods. In this context, herbal medicine has once again increased in importance as it can be used to provide effective, safe, and sustainable treatment for infections [1–4]. Plant extracts, rich in heterogeneous bioactive molecules, have been used in traditional medicine for centuries and are increasingly being utilized in novel pharmaceutical and biotechnological applications [5, 6]. Among the innovative approaches to improve the extraction and application of bioactive molecules, deep eutectic solvents (DESs) have emerged as a promising tool due to their unique properties and eco‐friendly nature. DES, as a type of ionic liquid, has been widely investigated in the past few years for their several potential applications in the branches of chemistry, biology, and environmental sciences [7, 8]. DES are usually formed by a mixture of a hydrogen bond acceptor (HBA), such as a sugar or salt, and a hydrogen bond donor (HBD), such as a carboxylic acid, amine, or phenol, resulting in a liquid solution with a lower melting point than its components. This property allows DES to exhibit various solvent‐like qualities, proving them to be an alternative to traditional solvents such as organic solvents and water [8, 9]. DES have been shown to have excellent solvent properties, such as high solubility, low toxicity, and biodegradability, which make them suitable for use in various applications, including biocatalysis, extraction, and drug development. The increasing interest in DES is also related to their potential to replace conventional solvents, offering a more sustainable and environmentally friendly solution for many industrial processes [8]. Plant extracts have wide applications in the pharmaceutical, cosmetic, and food industries due to their therapeutic, nutritional, and functional properties [2, 10, 11]. Plant extraction with DES is particularly important since it has the potential to solubilize a wide range of bioactive compounds such as polyphenols, terpenes, and alkaloids from plant materials like leaves, roots, and flowers. DES‐based extraction methods are superior to conventional solvents in terms of yield, selectivity, and speed. This leads to higher quality extracts with enhanced bioactivity and lower processing costs. Furthermore, DES are “green solvents” as they are typically derived from natural sources, biodegradable, and nontoxic, which reduces the environmental impact and health risks of the application of conventional solvent‐based extraction methods [8, 12, 13]. As interest in natural extracts continues to grow, the integration of DES into plant extraction is an important step toward sustainable and efficient extraction methods [8, 14]. *Althaea officinalis* (*A. officinalis*) or marshmallow is a perennial plant of the Malvaceae family that has been traditionally used in medicine for centuries due to its diverse range of therapeutic properties. *A. officinalis*, which grows naturally in Europe, Western Asia, and North Africa, has been used in the treatment of numerous diseases such as gastrointestinal diseases, respiratory diseases, and skin diseases [15]. The leaves and roots of the plant contain a complex mixture of bioactive molecules such as mucilages (∼5–11.6%), flavonoids (e.g., apigenin‐7‐glucoside, quercetin, catechin), phenolic acids (caffeic, ferulic, chlorogenic, syringic acids), tannins, coumarins, and triterpenoids, which are believed to contribute to its therapeutic properties [16–19]. The antimicrobial and anti‐inflammatory activities of the plant inhibit infection and reduce the severity of inflammation, thereby accelerating the wound‐healing process [20]. These properties make *A. officinalis* a promising candidate for the development of natural antimicrobials, especially in the context of increasing antibiotic resistance. Ultrasound‐assisted extraction (UAE) is an extraction technique that combines the processes of acoustic waves and solvent extraction to efficiently recover beneficial biomolecules from plants and other materials [8]. This innovative method uses the power of ultrasound waves to disrupt cellular structures, release bound compounds, and increase mass transfer rates, resulting in improved extraction yields and reduced amounts of solvent consumption [21, 22]. Overcoming the limitations of traditional extraction techniques, UAE offers a convenient approach for the isolation of bioactive compounds, such as polyphenols, proteins, and essential oils, for potential applications in food, pharmaceuticals, and cosmetics. This method has attracted much attention in recent years due to its many advantages, including reduced processing time, increased extraction efficiency, and environmentally friendly processing conditions. In the present research, the aim was to investigate the antimicrobial activity of aqueous, methanolic, and DES‐based extracts of *A. officinalis* against standard strains of bacteria and yeast species, as well as clinical isolates of *Candida* species (Figure 1). Investigating the antimicrobial activity of aqueous, methanolic, and DES‐based extracts of *A. officinalis* has the potential to develop natural antimicrobial agents, validate the practices of traditional medicine, and elucidate the possible applications of the plant in modern medicine, wound healing, and green biotechnologies.

## 2. Materials and Methods

### 2.1. Plant Material

The flowers of the *A. officinalis* plant were purchased from Hayar Teb Company, a supplier of medicinal herbs (Iran, Fars Province, Shiraz). The flowers were ground and stored at −20°C until analysis.

### 2.2. Chemicals

All chemical materials were of analytical grade. Ammonium acetate (AA), 1,1‐diphenyl‐2‐picrylhydrazyl (DPPH), methanol, Sabouraud Dextrose Agar (SDA), and Mueller‐Hinton Agar (MHA) plates were obtained from Merck Company (Darmstadt, Germany). RPMI‐1640 was purchased from Sigma‐Aldrich Company. Folin–Ciocalteu reagent was prepared from BDH Company. Lactic acid (LA) was from Rhone Poulenc (France). 3‐(4,5‐Dimethylthiazol‐2‐yl)‐2,5‐diphenyl tetrazolium bromide (MTT), Dulbecco’s modified Eagle’s medium nutrient mixture F‐12 (DMEM/F12), and fetal bovine serum (FBS) were purchased from Gibco, BRL (Eggenstein, Germany). Sodium carbonate, normal saline, and distilled water were available in the laboratory.

### 2.3. Preparation of DES

The DES was synthesized by heating AA and LA as the HBA and HBD components, respectively. The molar ratios of 1:1, 1:2, and 1:3 were examined for HBA and HBD, respectively. Among these, the ratio of 1: 2 resulted in a higher volume of extraction. Therefore, the extraction process was performed using the ratio of 1: 2 for AA and LA, respectively. The components of each ratio were combined in a tightly sealed flask and stirred at 50°C until a uniform and colorless liquid was achieved [8].

### 2.4. Extraction Process

The optimized parameters of the extraction process using DES solvent were selected according to the previous studies [7, 8, 23–25], with modifications made to further enhance the results. Briefly, the powdered dried flower (0.25 g) was mixed with 3 mL of DES solvent. The solvent contained 19.41% water to decrease viscosity. Subsequently, the extraction process was carried out using ultrasonic waves for 122 min at a temperature of 35°C. To increase contact between the solvents and the plant material, the tube was vortexed every 30 min.

For aqueous extraction, the same plant material was mixed with 3 mL of distilled water and subjected to UAE under identical conditions. Methanolic extraction followed the same protocol using 100% methanol (v/v) as the solvent. Finally, each of the extracts was filtered and stored at −20°C until analysis. UAE conditions (122 min, 35°C, 0.25 g sample in 3 mL solvent) were optimized based on previous studies and applied uniformly to all extracts (aqueous, methanolic, DES). The extraction conditions were adjusted using central composite design (CCD).

### 2.5. Total Phenolic Content (TPC)

The TPC for three phases, including aqueous, methanolic, and DES extracts, was measured using the colorimetric method with the Folin–Ciocalteu reagent [26]. For each extract, the procedure involved adding 0.78 mL of distilled water, 0.02 mL of the extract, and 0.05 mL of Folin–Ciocalteu reagent to a test tube, which was then vortexed for 1 minute. Subsequently, 0.15 mL of 20% sodium carbonate was added to each extract, and the mixture was vortexed again. The solution was allowed to stand in a dark place for 60 min at room temperature. The absorbance for each extract was measured at a wavelength of 750 nm using a microplate reader (Polar Star Omega, BMG LABTECH GmbH, Germany). TPC was calculated using a calibration curve of gallic acid as the standard compound (mg/g of dry weight). Following this, the yield of total polyphenol (YTP) was calculated using the following equation [27]:

(1) YTP=TPC×Vm,

where *V* (*L*) represents the extract volume and *m* (*g*) indicates the dry weight of *A. officinalis*, respectively.

### 2.6. Antimicrobial Activity

#### 2.6.1. Microorganisms

The antimicrobial properties of three phases including aqueous, methanolic, and DES extracts of *A. officinalis* against six standard strains of yeast fungi, including *Candida albicans* (CBS 562), *C*. *dubliniensis* (CBS 8501), *C. glabrata* (ATCC 90030), *C. tropicalis* (ATCC 750), *C. parapsilosis* (ATCC 4344), and *C. krusei* (ATCC 6258), as well as standard strains of bacterial species including, *Staphylococcus aureus* (ATCC 52268), *Escherichia coli* (ATCC 25922), and *Pseudomonas aeruginosa* (ATCC 27853), were tested. Additionally, five clinical azole‐resistant and five azole‐sensitive isolates of *C. albicans* were examined in this study [28]. The mentioned fungi and bacterial species were cultured on SDA and MHA, respectively, and then incubated for 24 h (at 32°C for fungi and 37°C for bacteria).

#### 2.6.2. Antimicrobial Activity Assay

##### 2.6.2.1. In Vitro Qualitative Study

The antimicrobial potential was assessed using a two‐step method. Initially, the disk diffusion test was employed as a screening method to identify the extracts with high antimicrobial potential against standard strains of Gram‐positive bacteria, Gram‐negative bacteria, and yeasts. Screening was performed following the disk diffusion method according to a protocol from the Clinical Laboratory Standards Institute (CLSI M44‐A2). Twenty‐four‐hour colonies, previously grown on MHA for bacteria and SDA for yeasts, were used to prepare the inoculum in sterile saline. The turbidity was adjusted to a 0.5 McFarland standard for uniformity. The suspension was used to inoculate 6‐cm‐diameter plastic Petri dishes with MHA media for bacteria and yeast. A total amount of 30 μL of the *A. officinalis* aqueous, methanolic, and DES extracts was placed on filter paper discs (5 mm diameter). The plates were then incubated for 18 h at 35°C ± 2°C for bacteria and 48 h at 32°C for the fungal strain. Antimicrobial activity was assessed by measuring the diameter of the growth inhibition zone, expressed in millimeters (mm) [29, 30]. The positive controls included antibiotic disks of ciprofloxacin (CIP, 5 μg) for the bacteria, and itraconazole (Itr, 100 μg) for the yeasts.

##### 2.6.2.2. In Vitro Quantitative Evaluation (MIC, MBC, and MFC)

The quantitative assessment was conducted using the broth microdilution method based on the Clinical and Laboratory Standards Institute (CLSI, protocol M27‐A3) to establish the minimum inhibitory concentration (MIC), minimum bactericidal concentration (MBC), and minimum fungicidal concentration (MFC) for all extracts tested against standard and clinical microbial strains. The inocula of tested microbial species were prepared from 24‐ h cultures. Microbial suspensions were adjusted to 0.5 McFarland standard turbidity, equivalent to a stock suspension of 1–5 × 10^6^ cells/mL for yeast fungi and 1–1.5 × 10^8^ cells/mL for bacteria. For determining antimicrobial activities, serial dilutions of the extract (0.06–128 μL/mL) were prepared in MHB for bacteria and RPMI‐1640 medium for yeast fungi, in 96‐well microtiter plates. Then, 100 μL of the working inoculum of tested fungi and bacteria were added to the microtiter plates and incubated in a humid atmosphere at 37°C for 24 h for bacteria and 32°C for 24–48 h for yeast fungi. The culture medium alone and the culture medium with inocula (yeast or bacteria) were adjusted as negative and growth controls, respectively. Each experiment was carried out in triplicate. After the incubation period, the presence of growth in 96‐well microtiter plates was compared with the growth control. The lowest concentration of the mentioned treatments, without visible growth, was considered as MIC. Additionally, 10 μL of medium from wells with no visible bacterial growth was transferred to MHA to determine the MBC, and 10 μL of medium from wells with no visible yeast fungi growth on SDA was used to specify MFC. The MBC and MFC values were assessed as the lowest concentrations producing less than 4 colonies (indicating 99.9% mortality of the microbes in the initial inocula). The positive controls were CIP for bacteria and Itr for yeasts [29, 30]. The DES solvent as a blank sample was evaluated for antimicrobial activity.

### 2.7. Activity of DPPH Radical Scavenging

The antioxidant capacity of the three phases, including aqueous, methanolic, and DES extracts of *A. officinalis,* was evaluated by the 2,2‐diphenyl‐1‐picrylhydrazyl (DPPH) free radical scavenging method. In this study, extract solutions were prepared at concentrations of 4, 8, 16, and 32 μL/mL. The assay was conducted by adding 100 μL of the DPPH ethanolic solution (0.3 mM) to the concentration samples in each well of 96‐well cell culture plates. DPPH solution served as a standard in this experiment. Subsequently, the samples were mixed and incubated in a dark environment for half an hour. Finally, the absorbance of the samples was measured using a UV–VIS plate reader at 517 nm (BMG Spectro Nano plate reader, Germany). The degradation of DPPH was calculated using the following equation:

(2) DPPH radical scavenging activity%=A0−AsampleA0×100,

where Asample is the absorbance of the (DPPH + ethanol + xerogel) and A0 is the absorbance of the control (DPPH + ethanol) [30, 31].

### 2.8. In Vitro Cell Culture Studies

The cytotoxicity of the three phases, including aqueous, methanolic, and DES extracts of *A. officinalis,* was quantitatively analyzed using the MTT test. The 3T3 mouse fibroblast cell line was cultured in DMEM/F12 medium containing 10% (v/v) FBS, 100 units/mL of penicillin, and 100 μg/mL of streptomycin in a humidified incubator at 37°C with 5% CO_2_. Sterilized extract concentrations of 8, 16, and 32 μL/mL were added to each well (3 replicates). No treatment was applied to one well, which served as a control group. At each time point (1, 2, and 3 days after cell seeding), the culture medium was removed, and 0.2 mL of MTT solution (0.5 mg/mL) was added to each well, incubated at 37°C for 4 h in a dark incubator. After that, the solution was discarded, and 0.1 mL of DMSO was added to each well to dissolve the formed formazan crystals. The absorbance of the samples was then read with a microplate reader (wavelength: 570 nm) from Biotech Instruments [32].

## 3. Results

### 3.1. FTIR Analysis

**Figure 2:**
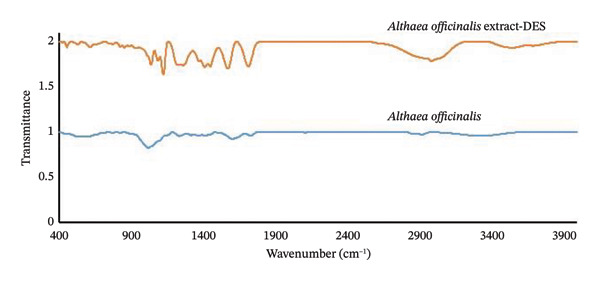
FTIR spectra of *A. officinalis* and *A. officinalis* DES extract.

FTIR spectroscopy is a versatile analytical technique that provides valuable information about the molecular structure of a sample. In the FTIR spectrum of the *A. officinalis* DES extract (Figure 2), a peak at 3530 cm^−1^ corresponds to the hydroxyl group and amine bonds present in the components of DES and the *A. officinalis* plant, which is also observed at 3330 cm^−1^ in the plant. The band at 2980 cm^−1^ is related to the C–H bonds in both the components of DES and the *A. officinalis* DES extract. The peak at 1714 cm^−1^ is associated with the carbonyl group of DES and the *A. officinalis*, which is seen at 1760 cm^−1^ in the plant. Peaks around 1570 cm^−1^ can be attributed to the symmetric stretching of carboxylate in AA (a component of DES) and the *A. officinalis*, with a shift observed at 1608 cm^−1^ in the *A. officinalis*. The absorption band at 1450 cm^−1^ is assigned to the bending of the C–H bonds of the methyl group in both the components of DES and the *A. officinalis*. Finally, the peak at 1041 cm^−1^ is associated with the stretching of the C–O bond in the components of DES extract and the *A. officinalis*, with a shift at 1025 cm^−1^ in the *A. officinalis*.

### 3.2. TPC

**Figure 3:**
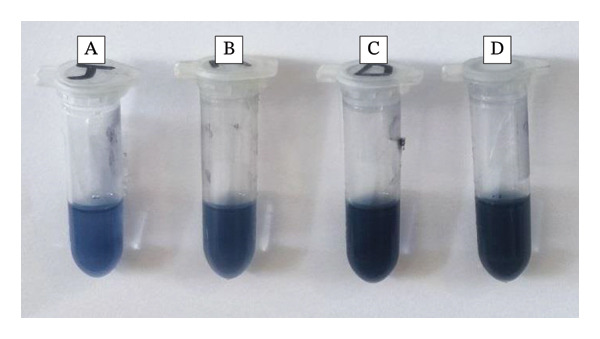
Analysis of total phenolic content for *A. officinalis* extracts: (a) aqueous, (b) methanolic, (c) DES, and (d) gallic acid.

Phenolics are a group of plant‐derived compounds that have been researched extensively for their anti‐inflammatory, antimicrobial, and antioxidant activities. The TPC in plant extracts refers to the total amount of phenolic compounds that comprise flavonoids, phenolic acids, and lignans. These compounds play an important role in protecting plants against environmental stresses and have also been associated with various human health benefits, including reducing the risk of chronic diseases such as cancer and neurodegenerative disorders. The TPC assay is often used as a marker of overall antioxidant capacity and potential bioactivity of plant extracts, which has considerable value for the development of natural products, functional foods, and medicinal plants [33, 34]. In the present study, the YTP of three *A. officinalis* extracts (aqueous, methanolic, and DES extracts) appears in Table 1 and Figure 3. The YTP values for the three extracts indicate that the DES extract (YTP = 8.9) is more effective than the aqueous (YTP = 4.1) and methanolic (YTP = 4.3) phases in phenolic compound extraction from *A. officinalis*. This indicates that the DES used in this study is a more efficient solvent for extracting phenolic compounds from the plant material. Variation in YTP among the three extracts can be attributed to the different solvation properties and interactions between the solvent systems and the phenolic compounds present in *A*. *officinalis*. The hydrogen bonding and electrostatic interactions between the DES components and the phenolic compounds may have enhanced the solubilization and extraction of the compounds, and thus affecting the YTP value. The polarity and solubilizing power of the solvents used may influence the extraction of phenolic compounds, where DES is more efficient than methanol and water.

**Table 1:** Total phenolic content and yield of total polyphenol values for *A. officinalis*.

Extract types	Yield of total polyphenol (mg GAE/g DW)^∗^	RSD (%)
Aqueous	4.1 ± 0.25	6.1
Methanolic	4.3 ± 0.41	9.5
DES	8.9 ± 0.40	4.5

*Note:* The data are presented as mean ± SD and relative standard deviation (RSD).

^∗^(mg GAE/g DW): Total phenol values were expressed regarding gallic acid equivalents.

### 3.3. Antimicrobial Result

#### 3.3.1. In Vitro Qualitative Study

**Figure 4:**
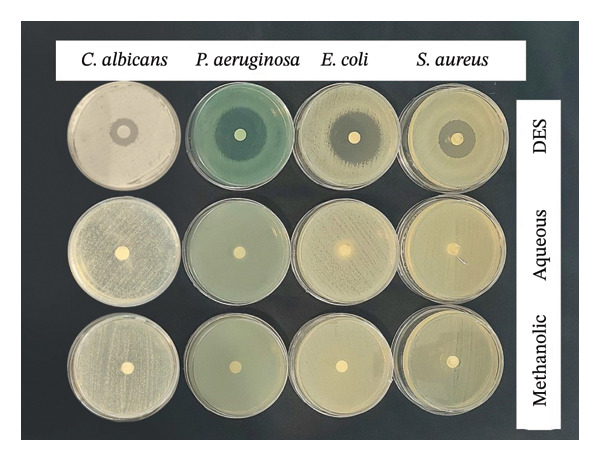
Inhibition zone of *C. albicans*, *P. aeruginosa*, *E. coli*, and *S. aureus* in the presence of different extracts of *A. officinalis*.

Using the disk diffusion test, the initial screening technique was designed to identify the antimicrobial potential against tested standard microbial strains. The results obtained under optimal conditions are depicted in Figure 4 and summarized in Table 2.

**Table 2:** The results of the disk diffusion test assay for the investigated Gram‐positive, Gram‐negative, and fungal standard strains.

**Fungi & bacteria standard strains**	**Organisms**	**ATCC/CBS**	**Different extracts of *A. officinalis***				
			**Aqueous**	**Methanolic**	**DES**	**RSD**	**Control (Itr & CIP)**

Fungi species	*C. albicans*	C (562)	0	0	15 ± 1	6.7	14
	*C. glabrata*	A (90030)	0	0	10 ± 1	10	13
	*C. dubliniensis*	C (8501)	0	0	10 ± 1	10	13
	*C. krusei*	A (6258)	0	0	16 ± 2	12.5	17
	*C. tropicalis*	A (750)	0	0	8 ± 1	12.5	15
	*C. parapsilosis*	A (4344)	0	0	19 ± 1	5.3	23

Bacteria species	*S. aureus*	A (52268)	0	0	30 ± 2	6.7	20
	*E. coli*	A (25922)	0	0	32 ± 1	3.1	23
	*P. aeruginosa*	A (27853)	0	0	35 ± 2	5.7	25

*Note:* The results are expressed as the diameter of the inhibition area (mm). The data are presented as mean ± SD and relative standard deviation (RSD). CBS: Centraalbureau voor Schimmelcultures; Itr: Itraconazole, CIP: ciprofloxacin.

Abbreviation: ATCC, American Type Culture Collection.

Overall, results regarding the antimicrobial activity of DES showed significant antibacterial (30–35 mm inhibition zone) and antifungal (8–19 mm inhibition zone) activities, while for methanolic and aqueous solvents, there was no activity. Overall, increased antimicrobial efficiency was observed for DES solvent tested against *P. aeruginosa* and *E. coli*, followed by *S. aureus*.

#### 3.3.2. In Vitro Quantitative Study

The disk diffusion test was used to evaluate the *A. officinalis* extracts for antimicrobial activity. The experimental outcomes from the qualitative study were confirmed by the quantitative assessment using the broth microdilution method, and they are summarized in Table 3.

**Table 3:** Antimicrobial effects of different extracts of *A. officinalis* on the fungi and bacteria standard strains based on the broth micro dilution method.

Fungi & bacteria standard strains	Organisms	ATCC/CBS	Different extracts of *A. officinalis*						
			Aqueous		Methanolic		DES		Control (Itr & CIP)
			MIC (μL/mL)	MFC/MBC (μL/mL)	MIC (μL/mL)	MFC/MBC (μL/mL)	MIC (μL/mL)	MFC/MBC (μL/mL)	
Fungi species	*C. albicans*	C (562)	G	—	16	32	8	16	0.5
	*C. glabrata*	A (90030)	G	—	32	—	16	32	0. 5
	*C. dubliniensis*	C (8501)	32	—	16	—	4	32	0.25
	*C. krusei*	A (6258)	G	—	16	—	8	16	0.25
	*C. tropicalis*	A (750)	32	—	4	32	2	8	0.25
	*C. parapsilosis*	A (4344)	G	—	8	—	4	8	0.5

Bacteria species	*S. aureus*	A (52268)	G	—	8	32	8	16	0.5
	*E. coli*	A (25922)	G	—	8	16	4	32	0.5
	*P. aeruginosa*	A (27853)	G	—	G	—	4	8	1

*Note:* CBS: Centraalbureau voor Schimmelcultures; Itr: Itraconazole, CIP: ciprofloxacin, G (> 128): No inhibition (MIC) or killing (MBC/MFC) was observed at the highest concentration tested.

Abbreviation: ATCC, American Type Culture Collection.

The antimicrobial activity of different extracts of *A. officinalis* (aqueous, methanolic, and DES) against various fungal and bacterial strains was determined using the broth microdilution method. The DES extract had the highest antimicrobial activity with lowest MIC and MFC/MBC values among aqueous and methanolic extracts. In case of fungal strains, lowest MIC values were achieved with the DES extract, particularly against *C. tropicalis* (MIC = 2 μL/mL) and *C. dubliniensis* (MIC = 4 μL/mL), while the aqueous extract was largely inactive, and the methanolic extract was weakly active.

The methanolic extract exhibited MIC values of 4 to 32 μL/mL for *Candida* species, while the DES extract demonstrated significantly lower MIC values (2–16 μL/mL) for the same standard strains. The MFC values of the DES extract were 8–32 μL/mL, indicating the fungicidal activity of this extract against all the tested *Candida* species. For *C. glabrata*, the antifungal activity of tested extracts (aqueous, methanolic, and DES) was reduced, with MIC values: > 128(G), 32 μL/mL, and 16 μL/mL, respectively. In addition to their antifungal properties, the DES and methanolic extracts exhibited antibacterial activity against the standard strains. Conversely, the aqueous extract was ineffective against all tested bacterial strains (*S. aureus*, *E. coli*, and *P. aeruginosa*), as evidenced by their growth.

The methanolic extract showed MIC values of 8 μL/mL for *S. aureus* and 8 μL/mL for *E. coli*, without affecting *P. aeruginosa*, whereas the DES extract showed exceedingly low MIC values (4–8 μL/mL) for all the bacterial strains used in the tests. Notably, *P. aeruginosa* was resistant to the methanolic and aqueous extracts but showed an MIC of 4 μL/mL for the DES extract. These findings suggest that the bioactive compounds found in *A. officinalis* in the DES formulation, have significant potential for antimicrobial applications.

Regarding the clinical isolates of *C. albicans*, the results revealed distinct patterns based on azole sensitivity (Table 4). The control (Itraconazole) demonstrated strong activity against azole‐sensitive isolates (MIC: 0.003–0.25 μL/mL) but was ineffective against azole‐resistant isolates (MIC: > 16 μL/mL). For azole‐sensitive isolates, the DES extract showed the most potent activity, with MIC values ranging from 0.06 to 16 μL/mL and MFC values ranging from 0.5 to 32 μL/mL. Notably, the DES extract has demonstrated the ability to inhibit and kill azole‐resistant *C. albicans* isolates, with MIC values ranging from 8 to 16 μL/mL.

**Table 4:** MIC and MFC values of the different extracts of *A. officinalis* against ten clinical isolates of *C. albicans*.

Azole sensitivity	Sample code	Different extracts of *A. officinalis*						
		Aqueous		Methanolic		DES		Control (Itr)
		MIC	MFC	MIC	MFC	MIC	MFC	
Clinical azole–sensitive	1	0.25	—	8	32	0.06	0.5	0.25
	2	0.25	—	2	4	0.06	1	0.003
	3	16	—	G	—	16	32	0.003
	4	0.25	—	G	—	8	16	0.25
	5	G	—	G	—	16	32	0.003

Clinical azole–resistant	1	G	—	G	—	16	32	> 16
	2	G	—	G	—	16	—	> 16
	3	G	—	G	—	16	—	> 16
	4	G	—	G	—	8	32	> 16
	5	G	—	G	—	8	16	> 16

These results highlight a remarkable therapeutic potential of the DES extract, suggesting its potential use in combating fungal and bacterial infections caused by tested species. The DES blank (AA and LA without plant material) was evaluated against all test strains and showed no antimicrobial activity and no inhibition zones, confirming bioactivity derives solely from *A. officinalis* extract.

### 3.4. Antioxidant

**Figure 5:**
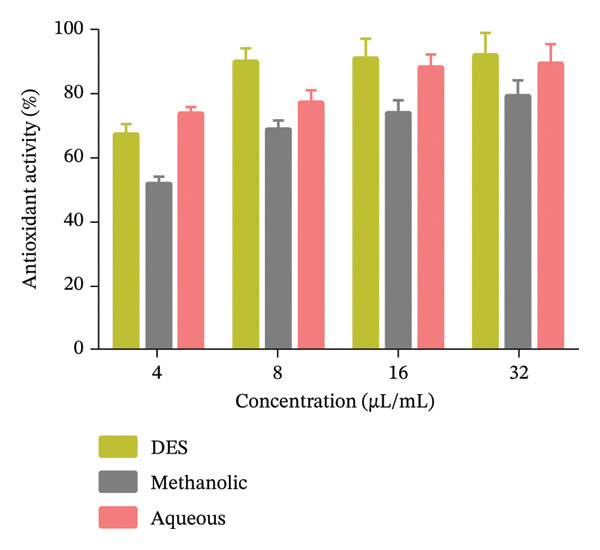
Antioxidant activity of the *A. officinalis* DES, methanolic, and aqueous extracts. The data are presented as mean ± SD.

Antioxidant activity of *A. officinalis* DES, methanolic, and aqueous extracts was measured, and the figure is presented as percentage antioxidant activity at various concentrations (Figure 5). The aqueous extract possessed mild antioxidant activity, with a gradual increase in activity as the concentration increased (73.9%–89.5%). DES extract exhibited the highest antioxidant activity (70.5%–92.1%) and showed dose‐dependent and significant improvement in its ability to scavenge free radicals. In contrast, the methanolic extract exhibited the lowest antioxidant activity (52.04%–79.3%), with only a slight increase in activity at higher concentrations.

Generally, at all tested concentrations, the DES extract was superior to the aqueous and methanolic extracts, indicating more potential for free radical neutralization. These results suggest that the extraction method significantly influences the *A. officinalis* antioxidant potential, with DES extract being the most effective in isolating antioxidant compounds. The differences in activity may be attributed to the variability in solubility and concentration of bioactive compounds such as phenolics and flavonoids in each extract. The DES extract exhibited the lowest IC_50_ (∼13 μL/mL), indicating superior antioxidant potency compared to aqueous (∼17 μL/mL) and methanolic (∼23 μL/mL) extracts. Low RSD (< 15%) confirms assay reproducibility (Table 5).

**Table 5:** IC_50_ values for DPPH radical scavenging activity of *A. officinalis* extracts.

Extract	IC_50_ (μL/mL)	RSD (%)
DES	∼13	< 10
Aqueous	∼17	< 12
Methanolic	∼23	< 15

### 3.5. Cell Viability Result

**Figure 6:**
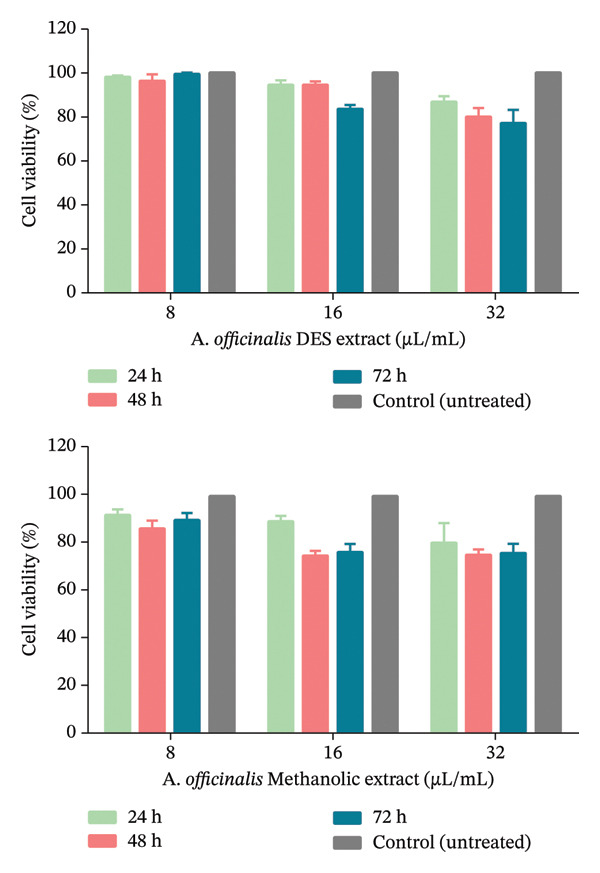
Effects of *A. officinalis* DES and methanolic extracts on cell viability of the 3T3 mouse fibroblast cell line.

The MTT assay was utilized to investigate the effects of *A. officinalis* DES, aqueous, and methanolic extracts on cell viability at concentrations of 8, 16, and 32 μL/mL over incubation periods of 24, 48, and 72 h (Figure 6). The aqueous extract of the *A. officinalis* exhibited 100% cell viability at all tested concentrations under investigation after 24, 48, and 72 h of incubation. The methanolic and DES extracts demonstrated time‐ and concentration‐dependent decreases in cell viability. For the DES extract, high cell viability (> 90%) was noted at 24 h across all tested concentrations (8–32 μL/mL). At 48 h, a dose‐related loss of viability to approximately 78% occurred at the maximum concentration (32 μL/mL); the trend in methanolic extract was comparable. At 24 h, cell viability remained above 85% even at the maximum concentration of the methanolic extract. By 48 h, the reduction in viability (∼75% at 32 μL/mL) was similar to that of the DES extract. The MTT assay results indicate that both *A. officinalis* DES and methanolic extracts show remarkably similar cell viability profiles, with no significant variation in their effects on cell viability (*p* value > 0.05). Interestingly, at concentrations showing antimicrobial, both extracts maintained cell viability in excess of 70% even after 72 h of exposure, indicating cell viability at clinically relevant doses. These findings suggest that both the DES and methanolic extracts have nearly identical and favorable cell viability profiles when used at concentrations effective against microbial pathogens, making them promising candidates for therapeutic applications.

## 4. Discussion

This study evaluated the antimicrobial, antioxidant, and cytotoxic activities of the *A. officinalis* extracts obtained using three different solvents: DES, methanol, and water. The results indicated significant differences in the bioactivity of the extracts, illustrating the effect of the extraction process on the yield and activity of bioactive compounds. DES extract produced the highest YTP of 8.9, significantly surpassing that of methanolic (YTP = 4.3) and aqueous (YTP = 4.1) extracts. This suggests that DES is a better solvent for recovering phenolic compounds from *A. officinalis,* owing to its unique solvation potential and ability to form hydrogen bonds with phenolic compounds. The raised TPC in DES extract is proportional to its increased bioactivity in subsequent tests.

Antimicrobial activity of the extracts was evaluated against fungal and bacterial isolates, including *Candida* species and Gram‐positive (*S. aureus*) and Gram‐negative (*E. coli* and *P. aeruginosa*) bacteria. The strongest antimicrobial activity was exhibited by the DES extract with high inhibition zones in the disk diffusion assay (30–35 mm against bacteria and 8–19 mm against fungi) and low MIC values in the broth microdilution assay. Interestingly, the DES extract was effective against azole‐resistant *C. albicans* clinical isolates with MIC values ranging from 8 to 16 μL/mL, suggesting its potential as a substitute treatment for resistant fungal infections. The significance of the DES extract lies in its effectiveness against clinical strains of *C. albicans*, particularly those that exhibit resistance to azole antifungals. Previous studies on *A. officinalis* using hydroalcoholic and methanolic extracts reported antibacterial activity against *S. aureus* (MIC = 330 μg/mL), *E. coli*, and *P. aeruginosa*, but generally weaker than the DES extract of the present study [10, 17]. Haghgoo et al. used the percolation technique for extraction and reported that the root extract of *A. officinalis* demonstrated antibacterial activity against *Lactobacillus acidophilus* and *Streptococcus mutans*, but this effect was less than that of CHX mouthwash (2%) and penicillin [35].

This is important given the rising antifungal resistance, which has become an imperative issue in the treatment of fungal infections. Resistance to azoles has primarily resulted from mutations in key genes such as ERG11, which is involved in ergosterol biosynthesis, a crucial part of the fungal cell membrane [36, 37]. The ability of the DES extract to inhibit these resistant strains suggests that it may target different pathways or mechanisms within the fungal cells and is a compound that should be included in antifungal therapy. The methanolic extract displayed moderate antibacterial activity against bacterial strains, with MIC values of 8 μL/mL against *S. aureus* and *E. coli* but it was ineffective against *P. aeruginosa*. The aqueous extract showed no antimicrobial activity against tested microbial strains. The results highlight the increased antimicrobial activity of the DES extract, an effect likely attributable to its higher concentration of bioactive compounds. These findings align with other studies, such as those by Kosakowska et al., who reported no antimicrobial effect for *Rhodiola rosea* aqueous and ethanolic extracts towards Gram‐positive and Gram‐negative bacteria, including *S. aureus* and *E. coli* [38]. The increased antimicrobial activity of the DES extract, due to its higher bioactive compound content, is also shown by studies like those of Grozdanova et al., who have indicated that DES significantly enhances the antimicrobial activities of plant extracts by destabilizing bacterial membranes and inhibiting biofilm formation [39]. The antimicrobial effectiveness of the DES extract against *P. aeruginosa* (MIC = 4 μL/mL) is particularly noteworthy since the pathogen already has innate resistance to many of conventional antibiotics. This observation is comparable to the findings of Olivares et al., who reported an MIC value of 1 μg/mL for imipenem stabilized in a DES extract against *P. aeruginosa* [40]. The ability of DES‐based extracts to inhibit such resistant pathogens highlights their potential as future alternative antimicrobial agents in the context of rising antibiotic resistance.

The antioxidant activity of the extracts was assessed by the DPPH radical scavenging assay. The maximum antioxidant activity (67%–92%) was found in the DES extract with dose‐dependent free radical scavenging activity. In contrast, the methanolic extract exhibited the lowest antioxidant activity (52%–79%), whereas the aqueous extract exhibited moderate activity (73%–89%). The superior antioxidant performance of the DES extract can be attributed to its higher phenolic content, which is well established to be responsible for the scavenging of free radicals. These findings show that the DES extract has significant potential for applications in oxidative stress‐related conditions. The results of this research are in agreement with previous studies on the antioxidant potential of plant extracts obtained using DES. For instance, past research demonstrated that DES‐based extraction methods significantly enhance the recovery of phenolic compounds from plant materials compared to traditional solvents like methanol and water (leading to higher antioxidant activity) [41]. Similarly, Zullaikah et al. reported that DES extracts of *Moringa oleifera* exhibited superior antioxidant activity due to their ability to solubilize of polar and nonpolar bioactive compounds, including flavonoids and phenolic acids [42]. The findings support the present work, highlighting the effectiveness of DES in extracting antioxidant compounds. In contrast, the low antioxidant activity of the methanolic extract aligns with the previous work studies. For example, Alara et al. determined that methanol was less effective than DES in extracting phenolic compounds from *Vernonia amygdalina* leaves, reducing antioxidant capacity [43]. The moderate antioxidant activity of the aqueous extract can be attributed to the limited water solubility of most phenolic compounds, as Ćujić et al. reported that aqueous extracts of dried *chokeberry* had lower phenolic content and antioxidant activity compared to DES‐based extracts [44]. The correlation between the antimicrobial and antioxidant properties of the DES extract suggests that the polyphenols and other bioactive compounds extracted using DES are multifunctional and play a role against microbial pathogens and oxidative stress.

The cytotoxicity of the DES, aqueous, and methanolic extracts was evaluated using the MTT assay on 3T3 mouse fibroblast cells. The aqueous extract showed 100% cell viability at all tested concentrations. The methanolic and DES extracts showed a concentration and time‐dependent reduction in cell viability. The similar safety of both extracts at bioactive concentrations suggests their potential as alternative antimicrobial agents with acceptable biocompatibility profiles relative to conventional antimicrobial agents. DES extract consistently outperformed the methanolic and aqueous extracts in all experiments, demonstrating the highest phenolic content, antimicrobial activity, antioxidant capacity, and cell viability. This is due to DES being able to properly extract bioactive compounds with excellent capacity to dissolve polar and non‐polar compounds. The methanolic extract was revealed antimicrobial and antioxidant activities but was less effective than the DES extract. The aqueous extract had the lowest bioactivity, with minimal antimicrobial and antioxidant effects and no cytotoxicity. This is due to the water insolubility of bioactive compounds.

Polyphenols, the principal bioactive constituents of *A. officinalis*, exert their antimicrobial action through multiple mechanisms. They can destabilize bacterial cell membranes, disrupt osmotic balance, and inhibit essential bacterial enzymes such as proteases and nucleases, thereby inhibiting critical metabolic processes and bacterial growth. Polyphenols can interfere with bacterial DNA and RNA synthesis, disrupting replication and transcription processes, and thereby inhibiting bacterial proliferation [10, 39].The use of DES not only enhances the extraction of polyphenols but also stabilizes their chemical structure, preventing degradation and improving their antimicrobial activity [39]. This is supported by studies such as those of Olivares et al., which demonstrated that natural deep eutectic solvents (NaDESs) can retain the activity of antibiotics by stabilizing their chemical structure, hence allowing for continuous inhibition of bacterial enzymes [40]. Similarly, Nystedt et al. indicated that NaDESs improved the antimicrobial effects of polyphenols through bacterial membrane destabilization and inhibition of biofilm [45]. The results of our study are further supported by Bedair et al., who demonstrated the antimicrobial efficacy of NaDESs [46]. The DES extract exhibited significant inhibition zones and presented very low MIC values, indicating remarkable antimicrobial activity. The modes of action may be related to destabilization of bacterial cell membranes and the disruption of osmotic balance, contributing to cell lysis. The acidic apparent pH of the DES extract acts to augment these actions by making it easier to destroy key proteins for microorganism survival.

## 5. Conclusion

The conclusions of the study highlight the essential role that extraction plays in identifying the bioactivity of *A. officinalis* extracts. DES extract was found to be most active, exhibiting higher antimicrobial, antioxidant, and cytotoxic activity compared to the methanolic and aqueous extracts. These findings suggest that DES‐based extraction is an efficient approach to extract bioactive compounds from *A. officinalis* and possesses vast scope for applications in the fields of pharmaceuticals, nutraceuticals, and cosmetics. More research is needed to identify the specific compounds and to establish extraction methods optimized for large‐scale processing.

## Author Contributions

Zahra Zareshahrabadi: project administration, conceptualization, funding acquisition, methodology, writing–original draft preparation, and writing–review and editing. Hossein Khodadadi: methodology, visualization, and investigation. Forough Karami: formal analysis, writing–original draft preparation, and investigation. Reza Ghasemi and Ahmad Vaez: methodology. Hasti Nouraei: resources and validation. Kimia Sahraeian: methodology.

## Funding

This study was supported by Shiraz University of Medical Sciences, 31917.

## Disclosure

This study was extracted from the thesis of Reza Ghasemi.

## Ethics Statement

This research was found to be in accordance with the ethical principles and the national norms and standards for conducting Medical Research in Iran and has been approved by research ethics (IR.SUMS.MED.REC.1404.037).

## Consent

No consent for publication was required.

## Conflicts of Interest

The authors declare no conflicts of interest.

## Data Availability

The data that support the findings of this study are available from the corresponding author upon reasonable request.
